# Pilot study of attentional retraining for postpartum smoking relapse

**DOI:** 10.3389/fpsyt.2023.1231702

**Published:** 2023-10-11

**Authors:** Ariadna Forray, R. Gwen Gunter-Riley, Caro Maltz, Andrew J. Waters

**Affiliations:** ^1^Department of Psychiatry, Yale School of Medicine, New Haven, CT, United States; ^2^Department of Medical and Clinical Psychology, Uniformed Services University of the Health Sciences, Bethesda, MD, United States

**Keywords:** attentional retraining, ecological momentary assessment, relapse prevention, perinatal, craving, smoking abstinence

## Abstract

**Introduction:**

Tobacco smoking is a leading cause of preventable death worldwide. The perinatal period provides a unique opportunity for intervention, as many smokers quit smoking during pregnancy but relapse postpartum. Novel relapse prevention interventions that reduce the burden of treatment attendance in this population are needed. Attentional retraining (AR) has been shown to reduce attentional biases toward smoking-related stimuli, a cognitive process implicated in smoking, AR has not been applied to perinatal smokers, and the effect of AR on craving and smoking is not clear. The goal of this study was to evaluate the delivery of AR for smoking cues in perinatal smokers utilizing a mobile intervention.

**Methods:**

This pilot study utilized Ecological Momentary Assessment (EMA) methodology delivered on a mobile device to examine the relapse process and evaluate the utility of AR in former smokers attempting to remain abstinent postpartum. AR (or Control Training) was administered to abstinent smokers (*N* = 17) for up to 2 weeks both before and after delivery.

**Results:**

All 17 participants completed the study. There was evidence that AR reduced attentional bias in the AR group (vs. Controls). There was no evidence that AR reduced craving. An exploratory analysis revealed that there was no evidence that AR reduced smoking during the study period.

**Discussion:**

AR using EMA methodology *via* a mobile device is feasible in perinatal smokers. Further research using larger samples is required to evaluate the utility of mobile AR in reducing craving and smoking.

## 1. Introduction

Pregnancy and the postpartum period present unique opportunities and challenges for the 17 million reproductive age female smokers in the US (1). Smoking in the mother is associated with increased risks for cancer, heart disease, and chronic pulmonary disease, as well as adverse pregnancy outcomes (2–5). The health effects of second-hand smoke on newborns, which include increased risk for respiratory and ear infections, sudden infant death syndrome, behavioral dysfunction and cognitive impairment, are also significant (6). Close to half of women who were smokers prior to conception are able to quit smoking in pregnancy (7), but nearly 50% relapse within 2 weeks (8) and 80% relapse within a year after delivery (9, 10).

Other than contingency management (11–13), effective treatments for smoking in postpartum women are limited, as noted by the 2019 Cochrane review covering 77 studies, 19 of which specifically addressed perinatal populations (14). Psychotherapeutic interventions are only modestly effective in this population (14–18). For example, while a motivational and problem solving based intervention for perinatal patients temporarily increased the maintenance of postpartum smoking abstinence, relapse rates increased over time diminishing the effect of the treatment (18). In addition, the efficacy and safety of pharmacologic treatments for smoking are not yet established in pregnant and postpartum women (15, 19, 20). Thus, new, efficacious behavioral interventions are needed for perinatal women.

To develop effective interventions to prevent postpartum smoking relapse, it is imperative to understand the factors and psychological processes that influence return to smoking following delivery. Negative affect, stress, and urges/cravings have been implicated in relapse (21, 22). The factors influencing relapse in perinatal populations, as reported by mothers, include stress or the presence of another smoker which may induce craving (8). Other studies have reported that second-hand smoke exposure (23) and depression (24) have a strong influence on postpartum smoking relapse. Ecological momentary assessments (EMA) provide repeated sampling of real-world events, as they are influenced by environmental and situational cues. The use of EMA facilitates the study of situational factors that may serve as predictors of smoking in real-time. EMA data can also capture how individuals are differentially affected by factors such as affect and craving (25).

Another factor that influences smoking is “attentional bias” (AB) to smoking cues. AB is defined as the tendency to automatically attend to and maintain attention on smoking cues, and may be causally related to craving and use/relapse (26–28). Empirical research has shown that lower levels of AB are associated with higher success rates of short-term abstinence in smokers attempting to quit (29). Thus, a reduction in AB may reduce the likelihood of attending to smoking-related cues that could provoke craving. AB can be reduced through attentional retraining (AR), in which modified cognitive tasks are used to train participants’ attention away from salient stimuli. For example, in the current context, AR seeks to train perinatal former smokers to automatically attend away from smoking cues and toward neutral cues, i.e., reduce AB. The effects of AR may transfer to real world stimuli, meaning that individuals undergoing AR would be less likely to attend to smoking cues in the environment, and therefore experience less cue-provoked craving. Both laboratory and field studies have demonstrated that AR can reduce AB toward smoking-related stimuli (28, 30).

AR has not been evaluated in perinatal smokers or perinatal former smokers. In a perinatal population, it may be useful to administer AR on a mobile device, given the promise of these methods in this population (31). In this randomized controlled pilot study, we tested the effect of AR of smoking cues administered on mobile devices, both prepartum and postpartum, in perinatal former smokers attempting to remain abstinent. We examined whether AR delivered on a smartphone can reduce AB to smoking-related stimuli and reduce craving for cigarettes. We also examined the effect of AR on smoking during the study period, and explored whether study phase (prepartum vs. postpartum) moderated the effect of AR on study outcomes.

## 2. Methods

### 2.1. Participants

Participants (*N* = 17) were recruited from the obstetrical clinics at Yale New Haven Hospital. Participants self-identified their race/ethnicity as: 9 Black, non-Hispanic; 4 Black, Hispanic; 2 White, Hispanic; 1 White, non-Hispanic, 1 other (West Indian). Inclusion criteria were: 1) a history of smoking 5+ cigarettes/day and having achieved abstinence by 32 weeks’ gestation; 2) aged 18 to 40 years; 3) able to speak and write English; 4) Edinburgh Postnatal Depression Scale (EPDS) score < 10. Exclusion criteria included: 1) current substance use (e.g., alcohol, marijuana); 2) current major depressive disorder, minor depression, or history of any of such in the last 6 months; 3) presence of an Axis I psychotic disorder; 4) plan to relocate out of the area; 5) imminent incarceration; 6) planned inpatient hospitalization during study period. All participants had to meet these eligibility requirements before they could be enrolled and randomized to either condition. Data collection took place between May 2014 and February 2015.

### 2.2. Study design

This was a double-blind randomized controlled pilot trial. Enrolled participants were assigned to one of the two study conditions through “urn” randomization to ensure relatively equal allocation between treatment group (AR) and control with respect to age and severity of nicotine dependence. Participants and investigators were blinded as to study condition.

### 2.3. Procedure

**Figure 1:**
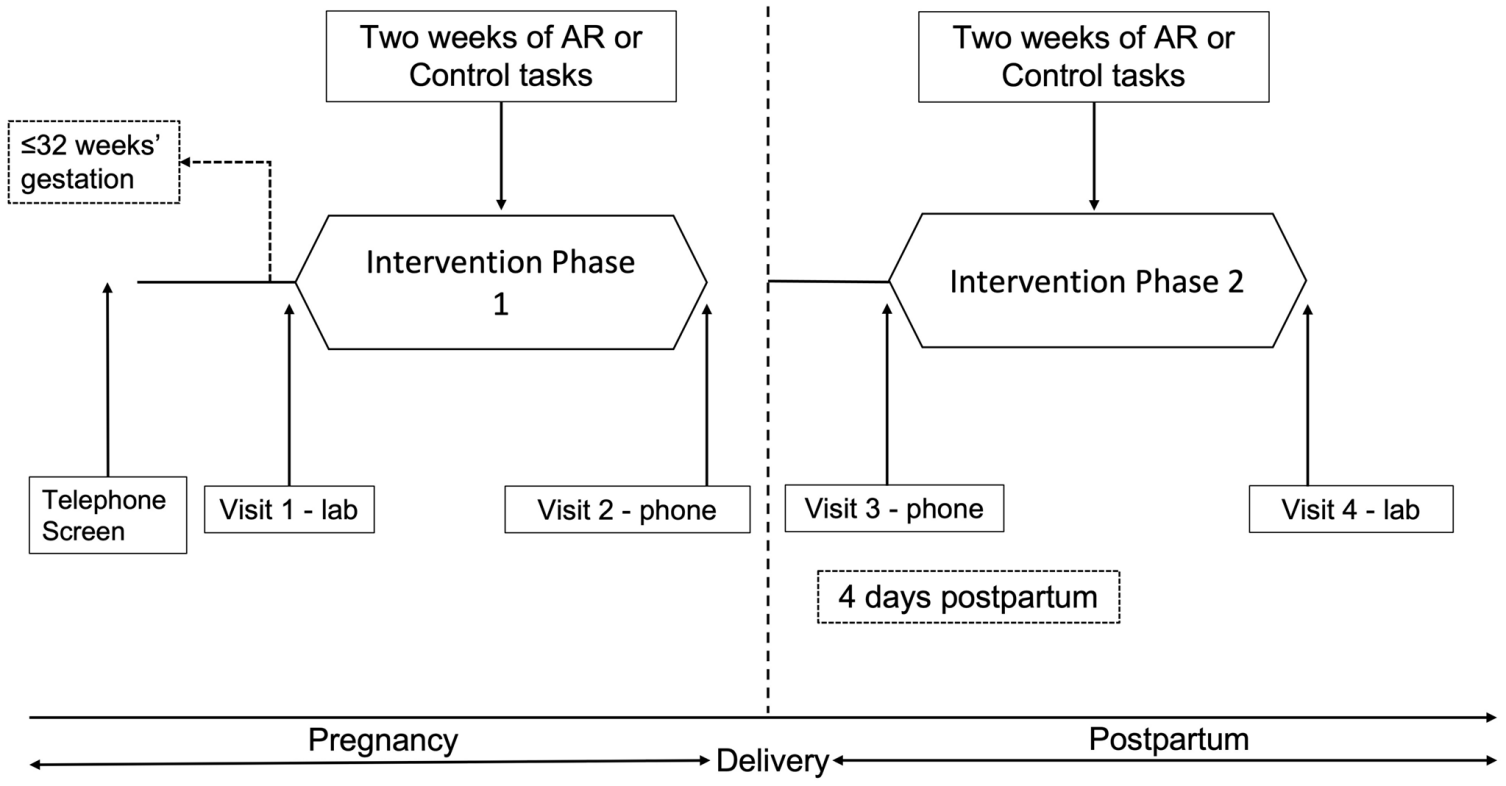
Procedures overview and timeline.

Figure 1 provides an overview of procedures. Pregnant patients awaiting a routine prenatal visit were invited to complete a screening survey to determine provisional eligibility after providing screening consent. Forty-one women were screened, 20 women were eligible, and 17 were enrolled in the study (Supplementary Figure S1). After screening, provisionally eligible women that were < 32 weeks’ gestation were followed until they reached 32 weeks’ gestation. Those who were still eligible for randomization at 32 weeks completed an intake interview (Visit 1) that included a review of study procedures and consent, computer administered intake assessments (Supplementary Table S1), collection of urine for toxicology and cotinine analysis, and breath sample for carbon monoxide analysis.

Following randomization participants were instructed to carry a smartphone (LG Fathom) as they went about their daily lives for 2 weeks (Phase 1). Participants were locked out of all functions other than the program and told they were to complete four random assessments (RAs) per day. To increase adherence, participants could use the “delay” feature if they needed to delay the task by 5 min (up to four times per day). A “suspend” option could be used if a participant needed to prevent the phone from presenting assessments for a specific time period. Participants could also “make-up” a training/assessment if they missed an RA or experienced technical difficulties.

After 2 weeks, participants were contacted *via* phone (Visit 2) and instructed that Phase 1 was completed and daily RAs were suspended. Approximately 4 days following delivery, participants began Phase 2, and were instructed *via* phone (Visit 3) to repeat the procedures from Phase 1. After 2 weeks they returned to the research clinic and completed Visit 4 assessments (Supplementary Table S1).

### 2.4. Measures

#### 2.4.1. Assessments

Measures administered at visits are listed in Supplementary Table S1 along with their psychometric properties (see Supplementary material S1). The Mini-International Neuropsychiatric Inventory 5.0.0 Clinician-Rated (MINI-CR) assessed the presence of a mood, psychotic, or substance use disorder (32). The Fagerstrom Test for Nicotine Dependence (FTND) assessed severity of nicotine dependence (33). The Parenting Stress Index (PSI)-Short Form (34) assessed parental stress. The Minnesota Nicotine Withdrawal Scale (M-NWS) (35) assessed symptoms of nicotine withdrawal. The Brief Questionnaire on Smoking Urges (BQSU) (36) assessed urges/craving for cigarettes “right now.” The Timeline Follow-Back (TLFB) assessed reported cigarette smoking for the prior week (37). All of these scales have been validated to suit research conducted in the U.S. and have been used in prior research with this population.

Breath carbon monoxide (CO) levels were used to confirm reports of abstinence. Participants’ expired breath CO level was measured with a Vitalograph Breath CO device (CO level of <4 ppm was used to indicate abstinence from smoking). The NicAlert® assay was used for the urine cotinine analysis which gives an output on a “0″ to “6″ ordinal scale; <3 was used to indicate abstinence from smoking.

#### 2.4.2. EMA procedures

EMA items, administered at RAs and make-up assessments, included the following: (1) overall mood and seven affect items (happy, calm, bored, sad, tense, irritable, tired) on a 7-point scale (1 = strongly disagree, 7 = strongly agree); (2) four items adapted from the Parenting Stress Index (“I feel I cannot handle things”; “I feel trapped by parenting”; “I feel overwhelmed by trying to meet my baby’s needs”; “Since the last assessment, my baby has been difficult to console”); (3) two items assessing recent smoking; (4) three items assessing general context; (5) two items assessing smoking context (“Right now, is anyone smoking around you? If so, who?”; “If you smoked a cigarette, was anyone else smoking around you at the time? If so, who?”); and (6) an item assessing craving for cigarettes a 7-point scale (as above) following exposure to a picture containing both smoking and non-smoking stimuli presented for 1 s, as described in Kerst & Waters. (30).

### 2.5. Intervention

At each assessment (RA or make-up), participants completed either a training task (AR or Control) (75% of RAs/make-ups), or a “standard” visual probe (VP) task (assessment of AB) (25% of RAs/make-ups).

#### 2.5.1. Standard VP task

In a standard VP task, a pair of pictures (e.g., one smoking-related and one neutral) is briefly presented (for 500 ms) simultaneously side by side on a computer screen. When the pictures disappear, a probe stimulus (e.g., a small dot) is presented in the location that had been occupied by one of the pictures (either on the left or the right), and participants are required to press a key as quickly as possible in response to the probe. AB for smoking-related cues is revealed by a faster response to a probe that replaces a smoking-related stimulus (vs. a neutral stimulus), since attention will have been allocated to the location where the smoking picture had been. Note that the standard VP task is an assessment of AB, and the assessment is not intended to change AB. The standard VP task was scored using typical procedures (see Supplementary material S2, S3).

#### 2.5.2. AR and control training conditions

On 3 of the 4 RAs scheduled each day, participants were scheduled to complete a training task (AR or Control), 160 trials each. On 1 of the 4 RA scheduled each day, participants were scheduled to complete the standard VP task (for assessment of AB), 80 trials each. During the standard VP task, the dot is equally likely to replace the neutral or smoking picture. Fifteen picture sets consisting of 20 picture pairs (one smoking-related and one neutral) each were used for the tasks. Images were displayed for 500 ms. One picture set was administered on each study day (days 0–14 in pregnancy and days 0–14 postpartum). For the AR condition the VP task was modified so the dot always replaced the neutral picture. In the Control condition the dot was equally likely to replace the smoking stimuli and the neutral stimuli ensuring no correlation between the picture type and dot location, thus avoiding training of attention. This type of control condition also ensures equivalency between the AR and control conditions in terms of task duration, motor practice and stimuli presented (38).

### 2.6. Data analysis

For both AB and craving, a linear mixed model (LMM) was used. Models included Group (AR vs. Control), Phase (Pre- vs. Postpartum), Day (within Phase) and, where appropriate, the Group x Day interaction. The primary analyses tested the main effect of Group and the Group x Day interaction. For Smoking, a binary outcome, a generalized linear mixed model was used (GLMM). Sample size considerations are reported in the Supplementary material S4. Data analysis was conducted with SAS version 9.4.

## 3. Results

### 3.1. Lab descriptive statistics

Seventeen subjects enrolled in the study, and all attended the final laboratory visit and reported completing at least some training (AR vs. Control). Fourteen subjects contributed EMA data. One subject returned the phone with the memory card removed (resulting in loss of EMA data), and EMA data from two other subjects could not be retrieved due to technical problems (see Supplementary material S5). Participant characteristics are summarized in Table 1. The mean age of participants was 27.88 years, and a high percentage self-identified as Black (76.5%). There were no significant Group (AR vs. Control) differences on age or race (Table 1). There were also no significant Group differences on the PSI, EPDS, MNWS, QSU-Brief, or CO (Supplementary Table S2).

**Table 1:** Baseline measures.

Assessment ↓	All	AR	Control			
	***N* = 17**	***n* = 9**	***n* = 8**			
	***Mean (SD) or n (%)***	***Mean (SD) or n (%)***	***Mean (SD) or n (%)***	***t/Chi Square***	***df***	***p***
Age	27.88 (*SD* = 4.92)	26.33 (*SD* = 4.18)	29.63 (*SD* = 5.37)	−1.42	15	0.18
Race/Ethnicity				4.78	2	0.31
Black	13 (76.5%)	8 (88.9%)	5 (62.5%)			
Puerto Rican	1 (5.9%)	0 (0.0%)	1 (12.5%)			
White	3 (17.7%)	1 (11.1%)	2 (25%)			
Hispanic Heritage				3.29	2	0.19
Puerto Rican and Dominican	1 (5.9%)	0 (0.0%)	1 (12.5%)			
Puerto Rican	4 (23.5%)	1 (11.1%)	3 (37.5%)			
None	12 (70.6%)	8 (88.9%)	4 (50.0%)			
Education (years)	11.71 (*SD* = 1.53)	11.11 (*SD* = 1.69)	12.38 (*SD* = 1.06)	−1.82	15	0.09
Employment				4.39	2	0.11
Full-Time	7 (41.2%)	4 (44.4%)	3 (37.5%)			
Part-Time	3 (17.6%)	0 (0.0%)	3 (37.5%)			
Not Working	7 (41.2%)	5 (55.6%)	2 (25%)			
Average Cigarettes Smoked/Day	11.00 (*SD* = 10.86)	9.78 (*SD* = 9.86)	12.38 (*SD* = 12.42)	−0.48	15	0.64
Age Smoking Initiation	15.65 (*SD* = 3.35)	16.44 (*SD* = 3.61)	14.75 (*SD* = 3.01)	1.04	15	0.31
Most Cigarettes Smoked per Day	17.18 (*SD* = 16.44)	12.22 (*SD* = 11.29)	22.75 (*SD* = 20.12)	−1.35	15	0.20
FTND	3.06 (*SD* = 2.84)	2.67 (*SD* = 3.16)	3.50 (*SD* = 2.56)	−0.59	15	0.56
Number of Pregnancies	4.00 (*SD* = 2.37)	4.33 (*SD* = 2.60)	3.63 (*SD* = 2.20)	0.60	15	0.98
Number of Births	1.88 (*SD* = 1.17)	1.89 (*SD* = 0.60)	1.88 (*SD* = 1.64)	0.02	15	0.98
Number of Children	1.47 (*SD* = 0.94)	1.67 (*SD* = 0.50)	1.25 (*SD* = 1.28)	0.90	15	0.38

Chi square statistics reflect pearson chi square statistics. Similar results are obtained using Fisher’s Exact Test.

### 3.2. EMA descriptive statistics

The 14 participants who provided EMA data completed 575 trainings/assessments in total, with 290 from participants in the AR group and 285 from participants in the Control group. In the Control group, 164 of the trainings/assessments were RAs and 121 were make-up. In the AR group, 93 of the trainings/assessments were RAs and 197 were make-up. In total, there were 257 RAs and 318 make-up trainings/assessments (see Supplementary material S6). On days on which participants completed at least one training or assessment, participants completed (either by an RA or make-up) a median of 73.86% of the expected number of trainings/assessments. Completion rate was not significantly associated with age (*p* = 0.40), number of children (*p* = 0.70), prior smoking rate (*p* = 0.77), FTND (*p* = 0.38), or EPDS score at baseline (*p* = 0.62).

### 3.3. Number of trainings

Across both prepartum and postpartum EMA phases, participants in the AR condition (*n* = 7) completed a mean of 28.29 (*SD* = 13.47) AR trainings, and Control participants (*n* = 7) completed a mean of 23.71 (*SD* = 12.63) Control trainings. The two groups did not differ in the number of trainings completed, *t* (12) =0.65, *p* = 0.52. Across phases, participants in the AR condition (*n* = 7) completed a mean of 8.14 (*SD* = 4.26) VP assessments, and Control participants (*n* = 7) completed a mean of 8.00 (*SD* = 4.58) VP assessments. The two groups did not differ in the number of VP assessments completed, *t* (12) =0.06, *p* = 0.95. Summary statistics on dependent variables by Group and Phase are presented in Supplementary Table S3.

### 3.4. AR effects

#### 3.4.1. Effect of AR on AB

**Figure 2:**
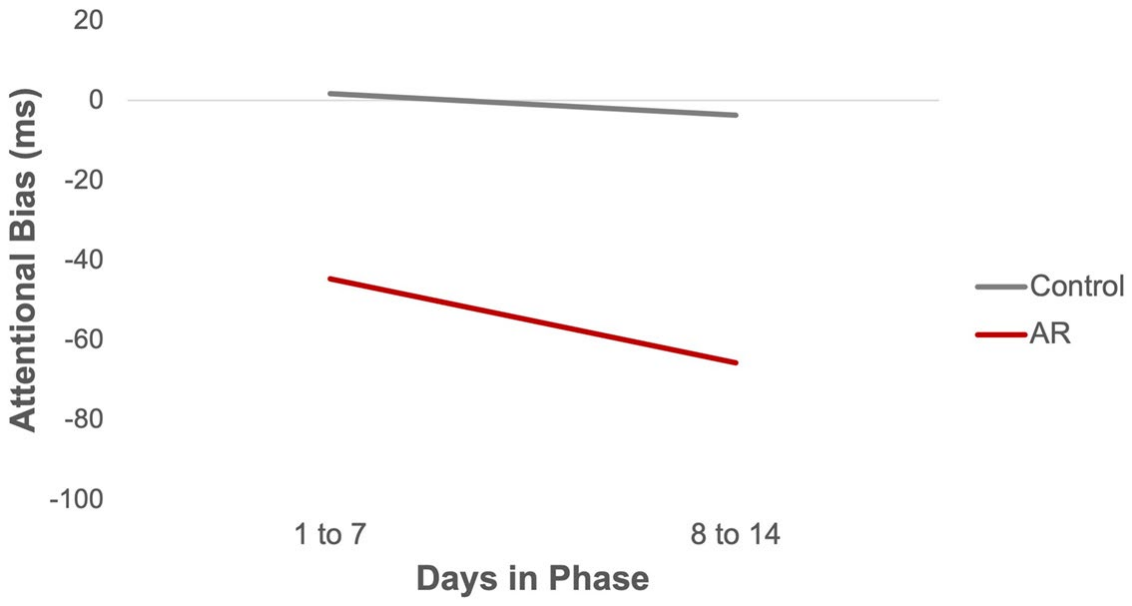
Attentional bias by group.

As shown in Table 2, AR significantly reduced AB. AB was about 49 ms lower in the AR group (*vs* Controls), corresponding to an effect size *r* = 0.66 when using the formula used by Kashdan et al. (39). Phase was not significant in the model (*p* = 0.69), meaning there was no evidence that AB changed across phases. The effect of AR on AB remained significant when controlling for recent smoking (*t* = −2.36, *p* = 0.04). To examine whether AB declined more over time in the AR group (*vs* Control) within Phases, a Group x Day interaction term was tested. Day within Phase, and the Group x Day interaction term, were included in a model that also included Group and Phase. When coefficients for Day were treated as fixed, the Group x Day interaction was significant (*PE* = −13.68, *SE* = 5.36, *t* = −2.60, *p* = 0.01), indicating that AB declined more over time in the AR group than Controls. When coefficients for Day were treated as random (i.e., allowed to vary over participants), the Group x Day interaction was not significant (*PE* = −22.63, *SE* = 13.00, *t* = −1.74, *p* = 0.11). Figure 2 presents summary data for AB as function of Group (AR vs. Controls) and days within phase (days 1–7, 8–14).

**Table 2:** LMM analyses.

			Numeric DV					Binary DV				
IV↓	DVs ↓	*n*	*df*	*PE*	*SE*	*F*	*P*	*Df*	*PE*	*SE*	*F*	*p*
Group (AR vs. Control)	Attentional Bias	271	1, 7.16	−48.54	20.52	5.59	0.04	.	.	.	.	.
Group (AR vs. Control)	Craving	575	1, 10.70	0.46	0.92	0.25	0.63	.	.	.	.	.
Group (AR vs. Control)	Smoking	565	.	.	.	.	.	1, 9.74	0.26	0.96	0.08	0.79

Data are results from LMMs (continuous outcomes) and GLMM (binary outcome). 7 AR subjects and 7 Control subjects contributed data to analyses. n = number of assessments. All models include Phase (parameter estimates for Phase not shown). df = Satterthwaite degrees of freedom, PE, parameter estimate, SE, standard error. Group coded as 0 = Control, 1 = AR.

#### 3.4.2. Effect of AR on craving

There was a non-significant main effect of AR on the EMA measure of craving (Table 2). Across all assessments, craving ratings were actually (non-significantly) higher in the AR group (vs. Control) (Supplementary material S3). Phase was not significant in the model (*p* = 0.26), meaning there was no evidence that craving changed across phases. We examined whether Craving declined more over time in the AR group (*vs* Control) within Phases by testing a Group x Day interaction term. Day within Phase, and the Group x Day interaction term, were included in a model that also included Group and Phase. The Group x Day interaction was not significant when coefficients for Day were treated as fixed (*PE* = 0.05, *SE* = 0.05, *t* = 1.00, *p* = 0.32), or random (*PE* = 0.11, *SE* = 0.11, *t* = 1.03, *p* = 0.34).

#### 3.4.3. Effect of AR on smoking

There was no evidence for a significant main effect of AR on smoking (Table 2). Phase was not significant in the model (*p* = 0.65), meaning there was no evidence that levels of smoking changed across phases. Regarding assessment of relapse, defined as any self-reported smoking during the study period, 3 participants (21.4% of 14 participants) reported no smoking during the entire study period, with abstinence confirmed with biochemical assessments, and 11 participants (78.6% of 14 participants) reported relapse. Two abstinent participants were in the AR group (28.6% of AR group) and 1 abstinent participant was in the Control group (14.3% of Control group).

### 3.5. Exploratory analyses

Exploratory analyses revealed that there was no evidence that the effect of Group was different in the two phases (see Supplementary material S7). In a supplementary analysis (see Supplementary material S8), we examined whether craving ratings declined over time. To be consistent with a previous study (28), analyses were conducted on the first two weeks of data collection (in this case, prepartum data). The effect of Day was significant for Craving, *F* (1, 8.06) = 5.92, *p* = 0.04, indicating that Craving declined over time. This model included Group and Day (Day was a level 1 numeric variable and coefficients for Day were treated as random). Additionally, when the Group by Day interaction was added to the model it was not significant, *F* (1, 7.62) = 0.21, *p* = 0.66. This means that the declines in Craving over time were not significantly different between the AR and Control groups.

## 4. Discussion

The main results of this pilot study of AR were as follows. First, there was evidence that AR reduced AB to smoking cues in perinatal women. Second, women in the AR group did not report significantly less craving than women in the Control group. Third, women in the AR group did not report a significantly lower rate of smoking than women in the Control group. Additional separate analyses by phase demonstrated that there was no evidence the effect of AR on study outcomes was different in the two phases (pre vs. postpartum).

Compared to Control training, there was evidence that AR reduced AB, as assessed by the VP task. AB was reduced about 50 ms in the AR compared to the Control group. This finding is consistent with other data suggesting that AR administered by the modified VP task can reduce AB as assessed by VP task [e.g., Robinson et al. (28)]. This suggests that AR can reduce AB in perinatal smokers when administered on a smartphone.

Although a significant effect of AR was observed, the following caveats should be noted. First, as noted earlier, the effect of Group was significant when all participants who provided EMA data (*n* = 14) were included in analyses (“intent-to-treat” analysis). However, the effect of Group was not significant in analyses restricted to individuals who were abstinent at baseline and who provided EMA data (*n* = 13). Therefore, more research is required to examine if the effect of AR in abstinent perinatal smokers is robust. Second, it was interesting that participants in the Control group did not exhibit significant AB. This is in contrast to data from participants who received Control training in previous studies (28, 30). However, one should bear in mind that the sample size in the Control group in the current study (*n* = 7) was smaller than sample sizes in other studies.

Compared to Control training, there was no evidence that AR reduced craving. This finding applied to craving assessed in the lab and field. This finding differed from those reported in a previous study (30). However, a null effect of AR on craving has also been reported in past research and thought to be due to the pictures not eliciting craving, thus compromising the ability of AR to reduce cued craving (28). It is also possible that in a natural, real-world environment, participants can become distracted and miss seeing the pictures, as the cues were presented for only 1 second.

Reported craving trended downward in pregnancy during Phase 1. Since there was no significant difference between the effect of Day in the two groups (AR and Control), this suggests a similar decline in craving in the two groups. Other researchers have reported that both AR and Control training can yield positive outcomes (40, 41). For example, Pettit et al. in an RCT of AR targeting pediatric anxiety found beneficial changes in both the AR and Control group (41). They speculated that both AR and attentional control training can reduce anxiety through repeated practice focusing, sustaining, and shifting attention which improves regulatory abilities improved in both groups. This suggests a different mechanism related to training flexible deployment of attention rather than a mechanism of change involving automatic attention allocation. These findings emphasize the need for further research regarding whether multiple cognitive mechanisms are affected during AR. However, given the absence of a no-treatment control group, these results should be treated with caution. Given that these finding were only seen in pregnancy, it is possible that declines in craving could have been independent of the AR or Control tasks, and due to other pregnancy related factors. For example, progesterone levels are at their highest in the late third trimester which is when participants engaged in the Phase 1. Progesterone is shown to decrease craving for nicotine in clinical studies (42, 43).

There was no significant effect of AR on smoking assessed on the smartphone, or on a biological measure of smoking assessed at the lab. Wiers and colleagues have argued that the effects of AR on drinking outcomes are more robust in clinical populations, who are generally strongly motivated to maintain abstinence, than in student samples or samples recruited online (44). Although our sample were recruited in a clinical context, and had made an attempt to abstain from smoking during pregnancy, there is still uncertainty regarding the level of motivation to remain abstinent after delivery. As noted, the relapse rate was high. Many mothers quit during the pregnancy for the health of the baby, but are not motivated specifically for their own health. Therefore, their level of motivation to remain quit after giving birth may greatly diminish, depending on where this motivation originated. It is possible that AR may only be effective in a selected sample of perinatal former smokers who are highly motivated to quit for good, rather than just “pausing” smoking during the prepartum period.

This study had a number of limitations. First, the sample size was small, which reduced power of analyses. The analytic sample size was further reduced by loss of data due to technical limitations. Therefore the findings, particularly the null effects of AR on craving and smoking, should be interpreted with caution pending further research with larger sample sizes. Nonetheless the data and findings may be useful for researchers for estimation of effect sizes and/or for use in meta-analyses. Second, due to participants’ extensive use of make-up assessments (rather than RAs) data from the study is likely less “random” than data from a study solely using random assessments. Use of make-up assessments reduces the generalizability of study findings and can potentially lead to bias in parameter estimates. Third, there were limitations regarding the assessment of AB. There was no baseline assessment of AB, meaning that it was not possible to determine whether the two groups differed at baseline. The study did not assess whether the effect of AR on attention generalized to different stimuli type (e.g., words) or to performance on a different attention bias task. Fourth, the use of a single item for craving of unknown reliability could be considered a limitation. Finally, while the main focus of the study was to examine AR in abstinent perinatal former smokers, there was evidence that one participant had smoked prior to randomization. Results should be interpreted in light of the fact that both abstinent and the non-abstinent individual were included in the intent-to-treat sample.

The study also had strengths. First, and most importantly, this was the first study to develop and administer an AR intervention for perinatal former smokers, a group at high risk of relapse. Second, another strength was the recruitment of an underserved minority population who are at risk of relapse and lifelong smoking.

Results from this study provide evidence that perinatal women can tolerate several days of training and that AR reduces AB in the field. Future research can build off the results of this study. It is possible that the effect of AR on outcomes is diluted by the presence of assessments administered in the field. Assessments were similar to Control trainings, and so future studies might manipulate the proportion of assessments to AR trainings in order to examine whether changes in proportion influence the effect of AR. As noted in the introduction, AR can be easily modified and has been modified for various health conditions and behaviors, such as healthier eating (i.e., train away from unhealthy food) and anxiety (i.e., train away from a perceived threat) (45, 46). Future research could examine the efficacy of training participants toward healthier behaviors or away from stress-related stimuli. Third, future research should evaluate factors that impact participant smoking behavior such as plans to breastfeed, and participants’ intention to remain quit or their motivation to quit. Lastly, examining the combined effect of AR with commonly used cessation treatments (e.g., CBT) is necessary to determine how much of an incremental effect AR can truly have in the real world.

## Data availability statement

The raw data supporting the conclusions of this article will be made available by the authors, without undue reservation.

## Ethics statement

The studies involving humans were approved by Yale University Institutional Review Board. The studies were conducted in accordance with the local legislation and institutional requirements. The participants provided their written informed consent to participate in this study.

## Author contributions

AF and AW contributed to the study’s conception, design, and data acquisition. AF, AW, and RG contributed to the analysis and interpretation of the data and the first draft of the manuscript. AW and RG performed the statistical analysis. CM wrote sections of the manuscript. All authors contributed to the article and approved the submitted version.
