# Supplementary material for: Pilot study of attentional retraining for postpartum smoking relapse
Source: Front Psychiatry. 2023 Oct 11;14:1231702. doi: 10.3389/fpsyt.2023.1231702 (PMC10603252; doi:10.3389/fpsyt.2023.1231702)
Supplement: Supplementary file 1 [file Data_Sheet_1.docx]

**Supplementary Materials**

**S1: Assessment Detailed Description**

The EPDS was used to assess depressive symptoms (Cox, Holden, & Sagovsky, 1987; Cox, Chapman, Murray, & Jones, 1996). It is designed specifically for use with pregnant and postpartum women. The scale is 10 items, has good internal consistency (Cronbach’s alpha = 0.87) and has been used in multiple different languages and different cultures ([Cox, Chapman, Murray, & Jones, 1996](#_ENREF_4); [Murray & Cox, 1990](#_ENREF_11)).

The Mini-International Neuropsychiatric Inventory 5.0.0 Clinician-Rated (MINI-CR) was used to determine the presence of a mood, psychotic or substance use (abuse and dependence) diagnoses ([Sheehan et al., 1997](#_ENREF_13)). The MINI-CR correlates highly with the SCID (0.43 to 0.90), and the Composite International Diagnostic Interview (CIDI) (0.36 to 0.82) but is shorter and easier to complete. It also has good inter-rater reliability, with kappa values greater than 0.90 across diagnoses.

The Fagerstrom Test for Nicotine Dependence (FTND) (Heatherton, Kozlowski, Frecker, Fagerstrom, 1991). It consists of six items that give a quantitative measure of the severity of nicotine dependence. The FTND is associated with biochemical indices (e.g., CO levels, plasma cotinine) of tobacco smoking ([Heatherton, Kozlowski, Frecker, & Fagerström, 1991](#_ENREF_6)). A review of studies that evaluated the reliability and validity of the FTND demonstrated it has moderate internal consistency, with Cronbach’s alpha coefficients ranging from 0.55-0.74, a correlation of 0.39 with cotinine verified smoking, and good test-retest reliability (0.87) ([Meneses-Gaya, Zuardi, Loureiro, & Crippa, 2009](#_ENREF_10); [Pomerleau, Carton, Lutzke, Flessland, & Pomerleau, 1994](#_ENREF_12)). For the sample sample in this study, Cronbach’s alpha was 0.71 (*N*=17), and 0.72 in the 13 women who identified as “Black Non-Hispanic” or “Black Hispanic”.

The Modified Kendler Social Support Interview (MKSSI) is a 21-item scale that measures social support from family and friends ([Kendler, Myers, & Prescott, 2005](#_ENREF_9)). Modified from the original Kendler Social Support Index, the MKSSI had a Cronbach's coefficient alpha of 0.86, indicating high reliability. Additionally, the MKSSI was significantly correlated to decreased odds of depression in the first trimester of pregnancy, which demonstrates external validity of this interview ([Spoozak, Gotman, Smith, Belanger, & Yonkers, 2008](#_ENREF_15)).

The Parenting Stress Index (PSI)-Short Form is an established measure of observed parent and child behaviors ([Abidin & Lutz, 1995](#_ENREF_1)). The PSI is a valid and reliable instrument that can assess a wide range of parenting behaviors in a single instrument, including: participant’s attachment to child, social isolation, sense of competence in the parenting role, relationship with spouse/parenting partner, role restrictions, and parental health. The PSI-SF has good test-retest reliability (*r*=0.75) and the correlation between the long and short form is also high (*r*=0.87) ([Haskett, Ahern, Ward, & Allaire, 2017](#_ENREF_5)). To reduce burden, five items from the PSI-SF were used in laboratory visits in the current study, which showed good internal consistency in this study (Cronbach’s alpha = 0.88).

The Minnesota Nicotine Withdrawal Scale (M-NWS) assessed DSM-IV-TR symptoms of tobacco withdrawal such as craving for nicotine, irritability, anxiety, difficulty concentrating, restlessness, headaches, fatigue, increased appetite, weight gain and insomnia ([Hughes & Hatsukami, 1986](#_ENREF_7)). The scale asks the participants to rate the presence of symptoms on a scale from “0” (not present) to “4” (severe). The MNWS has good validity and reliability as demonstrated by multiple studies showing it correlates as expected with health status, is predictive of withdrawal symptoms on subsequent cessation, and has internal reliability estimates above 0.70 ([Al-Mrayat, Okoli, Studts, Rayens, & Hahn, 2019](#_ENREF_2); [Joseph et al., 2005](#_ENREF_8))

The Brief Questionnaire on Smoking Urges (BQSU) is a reliable 10-item scale that is sensitive to nicotine deprivation (Bell, Taylor, Singleton, Henningfield, & Heishman, 1999; Morgan, Davies, & Willner, 1999). In the Study 1 dataset, Cronbach’s alpha = .89 at the enrollment visit and Cronbach’s alpha = .99 at the study completion visit.

The Timeline Follow-Back (TLFB) assessed reported smoking for the prior week ([Sobell & Sobell, 1992](#_ENREF_14)). This self-report assesses both the frequency and quantity of substance abuse using a visual calendar. While it used to assess use of various substances, it seems to produce valid estimates frequency and amount of tobacco smoking ([Collins, Eck, Torchalla, Schröter, & Batra, 2009](#_ENREF_3)).

**S2: Visual Probe Task Data**

Reaction times for incorrect responses were discarded, and RTs < 100 ms were also discarded. To reduce the influence of RT outliers, the median RT for probes that replace neutral and smoking pictures was computed. Attentional bias was computed as the median RT on probes that replace neutral pictures minus median RT on probes that replace smoking pictures. Greater attentional bias is indicated by faster response times on probes that replace smoking stimuli than probes that replace neutral stimuli. Bias scores were computed from all available data from which an attentional bias score can be computed. One extreme bias score was excluded (>5 *SD*s from overall mean). Average error rates on the tasks were low (*M*=4.45% errors, *SD*=10.54), but there were some trainings/assessments with high error rates, perhaps suggesting inattention. Bias scores with >40% errors were discarded (4% of scores) for primary analyses as this performance was not better than chance performance.

**S3: Stimuli for VP Tasks**

Pictures for the AR/control intervention and the VP task were selected from pictures using methods described in Robinson et al. (2017). The pictures included images of African Americans holding cigarettes, pictures of menthol cigarette packages, and brands frequently consumed by African Americans such as Newport (Glasser et al., 2016). We also included pictures of cigarette-related objects including lighters and ashtrays. The final pool of pictures included Caucasians, African Americans, and other races/ethnicities. Smoking objects generated for African American stimuli emphasized brands that African Americans smoke (Newport, Kools), whereas those for Caucasians emphasized other brands (e.g., Marlboro). Note that the pictures were not tailored to perinatal women in any way. That is, they were not generated from images that are representative of environments of perinatal (former) smokers. In addition, in Study 2 five participants endorsed ethnic heritage as “Puerto Rican and Dominican” or “Puerto Rican” and no attempt was made to include pictures corresponding to these ethnic categories.

From this final pool of pictures, “smoking object”, “neutral object”, “smoking human”, and “neutral human” pictures were selected. That is, each smoking picture was either a picture of a smoking object (such as a cigarette or pack of cigarettes) or a picture of a human (male or female) who was smoking. Correspondingly, each neutral picture was either a picture of a neutral object (such as a stapler) or a picture of a human (male or female) who was *not* smoking. Therefore, the pictures tended to be “simple”, which seemed appropriate given their small size on the smartphone screen.

From the resulting picture pairs, a number of picture lists were generated, each containing 20 pictures: 5 “smoking object”; 5 “neutral object”; 5 “smoking human”; 5 “neutral human”. The “smoking object” and “neutral object” pictures were randomly paired together. Likewise, the “smoking human” and “neutral human” pictures were randomly paired together. The ten picture pairs were organized so that half the time the smoking picture (object or human) would appear on the left side of the screen, and half the time it would appear on the right. The picture pairs were used to create 160 trials for AR/Control trainings and 80 trials for the standard VP assessments.

For further detail on the task stimuli and procedures, please see Robinson et al. (2017).

**S4: Sample Size and Power Considerations**

The study examines the effect of a manipulated level 2 variable (Group) on level 1 variables (e.g., Attentional Bias, Craving). This is therefore a 2 → 1 design. An a priori power analysis was conducted. Assuming 17 participants were randomized to treatment conditions and completed ~1008 trainings/assessments (which assumes 16 participants provide EMA data for an average of 3 weeks, with an average of 3 trainings/assessments per day), and an *ICC* of 0.1 (or 0.3), the study had power = .80 to detect a main effect of Group in the population, *d*, of 0.48 (0.80) for craving (which was recorded at every assessment). Power for attentional bias would be lower, as there were fewer assessments. If the *ICC* were greater (closer to 1) power would be lower. The power to detect interactions (e.g., Group x Day) is more complex to estimate, and is not presented here.

**S5: EMA Contributors vs. Non-Contributors**

As noted above, of the 17 participants who enrolled in the study 14 participants (82.4%) contributed EMA data to the study, and 3 participants (17.6%) did not contribute EMA data. Two Non-Contributors were in the AR group (22.2% of AR group), while 1 Non-Contributor was in the Control group (12.5% of Control group). The EMA Contributors self-identified as 78.5% Black, 7.1% Puerto Rican, and 14.3% White, Hispanic, and the Non-Contributors self-identified as 66.7% Black and 33.3% White, Non-Hispanic. For EMA Contributors and Non-Contributors respectively, the mean age was 27.86 (*SD*=5.33) and 28.00 (*SD*=3.00). The mean level of education was 11.57 years (*SD*=1.65) and 12.33 years (*SD*=0.58) respectively. EMA Contributors smoked an average of 11.36 per day (*SD*=11.78) with a mean FTND score of 3.57 (*SD*=2.85), whereas Non-Contributors smoked an average of 9.33 cigarettes per day (*SD*=6.03) with a mean FTND score of 0.67 (*SD*=1.15). EMA Contributors reported a mean number of 4.21 pregnancies (*SD*=2.58), and Non-Contributors reported a mean number of 3.00 pregnancies (*SD*=0.00).

**S6: EMA Descriptive Statistics**

The average interval between trainings/assessments for those that occurred on the same day as the previous assessment (*n*=388 assessments) was 2.59 hours (*SD*=2.76). In the AR group (*n*=198 assessments), the mean interval was 2.30 hours (*SD*=3.05) and in the control group (*n*=190 assessments), the mean interval was 2.90 hours (*SD*=2.40).

Regarding time of day, 22.6% of trainings/assessments were completed between 4.00 AM and Noon (“AM”), 45.7% completed between Noon and 7.00 PM (“PM”), and 31.7% were completed between 7.00 PM and 4.00 AM (“night”). Similar proportions occurred across groups. More specifically, in the AR group 22.8% were completed in the AM, 43.4% were completed in the PM, and 33.8% were completed at night. In the Control group 22.5% were completed in the AM, 48.1% were completed in the PM, and 29.5% were completed at night.

For the AR group the mean duration of completed AR trainings was 8.98 minutes (*SD*=3.94). For the Control group the mean duration of completed Control trainings was 8.74 minutes (*SD*=2.23). For the AR group the mean duration of completed assessments that included the VP assessment was 5.49 minutes (*SD*=2.84). For the Control group the mean duration of completed assessments (with the VP assessment) was 6.60 minutes (*SD*=2.77). Note that trainings were longer than the assessments because they contain more trials.

Regarding Phase (prepartum vs postpartum), thirteen participants (6 AR, 7 Control) contributed data (396 trainings/assessments; 170 from AR participants and 226 from Control participants) from the prepartum phase, 11 (7 AR, 4 Control) contributed data from the postpartum phase (179 trainings/assessments; 120 from AR participants and 59 from Control participants), and 10 participants (6 AR, 4 Control) contributed data from both phases.

**S7: Exploratory Analyses**

***Effect of AR at Prepartum and Postpartum phases***

An exploratory aim for this study was to examine whether study phase (prepartum vs. postpartum) moderated the effect of AR on study outcomes. In order to examine whether there were differences in the effect of Group in the two phases, analyses were conducted for each phase separately.

The prepartum phase was examined first. When craving was entered as the DV, there was a non-significant main effect of Group, *F* (1, 8.57)=0.19, *p*=.67. When smoking was entered as the DV, there was also a non-significant effect of Group, *F* (1, 8.69)=0.14, *p*=.72. Lastly, when attentional bias was entered as a DV, there was also a non-significant effect of Group, *F* (1, 7.93)=3.32, *p*=.11. The results for the postpartum phase were as follows. When craving was entered as the DV, there was a non-significant main effect of Group, *F* (1, 8.28)=0.42, *p*=.54. When smoking was entered as the DV, there was also a non-significant effect of Group, *F* (1, 8.68)=0.14, *p*=.72. Lastly, when attentional bias was entered as a DV, there was also a non-significant effect of Group, *F* (1, 7.13)=0.01, *p*=.93. In sum, analyses indicate there was no evidence that the effect of Group was different in the two phases. A formal moderation analysis was not conducted due to the complexity of multilevel moderation analyses involving a level 1 variable (Phase) that differed across subjects (e.g., some subjects only had data from a single Phase), and the small sample size.

**S8: Supplementary Analyses**

***Analysis of Lab Data***

As shown in Table S4, using ANOVA or ANCOVA, there were no significant effects of Group on post-treatment measures, including measures of craving (QSU-brief) and smoking (expired CO in breath).

***Analysis of Abstinent Subset***

Examination of descriptive statistics revealed that 1 woman (AR condition) had breath CO levels above the threshold pre-treatment (at enrollment), indicating the presence of recent smoking prior to data collection. Therefore, additional analyses were performed on the 16 participants (13 who provided EMA data) with no evidence of recent smoking so as to more clearly examine data relevant to relapse prevention.

When attentional bias was entered as a DV, the main effect of Group was marginally significant, *F* (1, 6.56)=4.87, *p*=.07. When craving was entered as the DV, the effect of Group was not significant, *F* (1, 9.87)=0.04, *p*=.84. When smoking was entered as the DV, the effect of Group was not significant, *F* (1, 9.16)=0.01, *p*=.92.

***Analysis Controlling for Baseline Measures***

Additional analyses were conducted controlling for baseline measures of dependent variables. This was not possible for attentional bias, as no assessment of attentional bias was taken at baseline. For Craving, in a model containing baseline BQSU, Phase, and Group, the main effect of Group was not significant (*PE*=0.02, *SE*=0.50, *t*=0.05, *p*=.96). For Smoking, in a model containing baseline FTND, Phase, and Group, the main effect of Group was not significant (*PE*=0.34, *SE*=0.61, *t*=0.55, *p*=.60).

***Additional Analysis of Attentional Bias Data***

The analysis of attentional bias used all available data from which an attentional bias score can be computed. For the AR group, an attentional bias score can only be computed from the VP assessment. For the Control group, an attentional bias score can be computed from the VP assessment and from Control trainings (which have the same structure as trainings but have more trials). For the Control group, attentional bias assessed in the Control trainings and the VP assessment were of similar magnitude (*M*=-2.34 ms, *SD*=122.48 and *M*=0.79 ms, *SD*=65.23 respectively, and did not differ significantly, *F* (1, 214)=0.03, *p*=.87). However, if the effect of AR on attentional bias was restricted to VP assessments, then the effect of Group was no longer significant, *PE*=-43.17, *SE*=36.81, *F* (1, 2.8)=1.38, *p*=.26. Note, however, that the parameter estimate, an unstandardized measure of effect size, is of comparable magnitude to the parameter estimate presented in Table 2.

As indicated in Table S3, there was no evidence that the Control group exhibited significant attentional bias. For example, when computing a mean attentional bias score for each Control subject (*n*=7) and then computing a mean of these subject means, the mean attentional bias was 1.47 ms (*SD*=10.03), which was not significantly different from 0 using a 1-sample *t*-test, *t*(6)=0.39, *p*=.71.

**References**

Abidin, R., & Lutz, F. L. (1995). *Parenting Stress Index: Professional Manual*

Al-Mrayat, Y. D., Okoli, C. T. C., Studts, C. R., Rayens, M. K., & Hahn, E. J. (2019). The psychometric properties of the minnesota tobacco withdrawal scale among patients with mental illness. *Biological Research for Nursing, 22*(2), 247-255.

Collins, S. E., Eck, S., Torchalla, I., Schröter, M., & Batra, A. (2009). Validity of the timeline followback among treatment-seeking smokers in Germany. *Drug and Alcohol Dependence* 164-167.

Cox, J. L., Chapman, G., Murray, D., & Jones, P. (1996). Validation of the Edinburgh Postnatal Depression Scale (EPDS) in non-postnatal women. *Journal of Affective Disorders, 39*(3), 185-189.

Glasser, A. M., Johnson, A. L., Rath, J. M., Williams, V. F., Vallone, D. M., Villanti, A. C. (2016). Tobacco Product Brand Preference among US Young Adults, 2011–2014. *Tobacco Regulatory Science*, 2, 44–55.

Haskett, M., Ahern, L., Ward, C., & Allaire, J. (2017). Factor structure and validity of the Parenting Stress Index-Short Form *Journal of Clinical Child and Adolescent Psychology, 46*(1), 170.

Heatherton, T. F., Kozlowski, L. T., Frecker, R. C., & Fagerström, K. O. (1991). The Fagerström Test for Nicotine Dependence: A revision of the Fagerström Tolerance Questionnaire. *British Journal of Addiction, 86*(9), 1119-1127.

Hughes, J. R., & Hatsukami, D. (1986). Signs and symptoms of tobacco withdrawal. *Archives of General Psychiatry, 43*(3), 289-294.

Joseph, C., Bushmakin, A., Baker, C., Merikle, E., Olufade, A., & Gilbert, D. (2005). Revealing the multidimensional framework of the Minnesota Nicotine Withdrawal Scale. *Current Medical Research and Opinion, 21*(5), 749-760.

Kendler, K. S., Myers, J., & Prescott, C. A. (2005). Sex differences in the relationship between social support and risk for major depression: A longitudinal study of opposite-sex twin pairs. *The American Journal of Psychiatry, 162*(2), 250-256.

Meneses-Gaya, I. C., Zuardi, A. W., Loureiro, S. R., & Crippa, J. A. (2009). Psychometric properties of the Fagerstrom Test for Nicotine Dependence. *Jornal Brasileiro de Pneumologia, 35*(1), 73-82. doi:10.1590/s1806-37132009000100011

Murray, D., & Cox, J. L. (1990). Screening for depression during pregnancy with the Edinburgh Depression Scale. *Journal of Reproductive and Infant Psychology, 8*(2), 99-107.

Pomerleau, C. S., Carton, S. M., Lutzke, M. L., Flessland, K. A., & Pomerleau, O. F. (1994). Reliability of the Fagerstrom Tolerance Questionnaire and the Fagerstrom Test for Nicotine Dependence. *Addictive Behavior Reports, 19*(1), 33-39. doi:10.1016/0306-4603(94)90049-3

Robinson, C. D., Muench, C., Brede, E., Endrighi, R., Szeto, E., Sells, J. R., Lammers, J. P., Okuyemi, K. S. & Waters, A. J. (2017). Effect of Attentional Retraining on Cognition Craving, and Smoking in African American Smokers. *Psychology of Addictive* *Behaviors*, 31, 636-646.

Sheehan, M., Sheehan, D., Lecrubier, Y., Harnett Sheehan, K., Janavs, J., Weiller, E., & Dunbar, G. (1997). The validity of the mini international neuropsychiatric interview (MINI) according to the SCID-P and its reliability. *European Psychiatry, 12*(5), 232-241.

Sobell, L. C., & Sobell, M. B. (1992). *Timeline Follow-back: A technique for assessing self-reported alcohol consumption*. New Jersey: Humana Press.

Spoozak, L., Gotman, N., Smith, M. V., Belanger, K., & Yonkers, K. A. (2008). Evaluation of a social support measure that may indicate risk of depression during pregnancy. *Journal of Affective Disorders, 114*(1), 216-223.

Table S1: Telephone/Lab Assessments

|  |  | **Phase 1: Pregnancy** | | **Phase 2: Postpartum** | |
| --- | --- | --- | --- | --- | --- |
| **Measure** | **Screening** | Visit 1 | Visit 2 | Visit 3 | Visit 4 |
|  | <32 wks | 32 wks | End Phase 1 | Start Phase 2 | End Phase 2 |
| Screening form | x |  |  |  |  |
| MINI International Diagnostic Interview |  | x |  |  |  |
| FTND |  | x |  |  |  |
| MKSSI |  | x |  |  |  |
| Urine toxicology |  | x |  |  | x |
| TLFB |  | x |  |  | x |
| Breath CO |  | x |  |  | x |
| Urine cotinine |  | x |  |  | x |
| EPDS | x |  |  |  | x |
| PSI |  |  |  |  | x |
| MNWS |  | x |  |  | x |
| QSU-brief |  | x |  |  | x |
| Phone check-in |  |  | x | X |  |

Table Note: MINI = Mini-International Neuropsychiatric Inventory; FTND = Fagerstrom Test for Nicotine Dependence; MKSSI = Modified Kendler Social Support Inventory; PSI = Parenting Stress Index; EPDS = Edinburgh Postnatal Depression Scale; CO = Carbon Monoxide; TLFB = Timeline Follow-Back; MNWS = Minnesota Nicotine Withdrawal Scale; QSU-brief = Brief Questionnaire for Smoking Urges. As shown, no assessments pertinent to the Study 2 were assessed at Visit 2 or Visit 3.

Table S2: Lab Assessments Summary Statistics

| **Group ↓** | **Assessment ↓** | **Screening** | **Enrollment** | **Study Completion** |
| --- | --- | --- | --- | --- |
|  |  |  | Visit 1 | Visit 4 |
|  |  | Phone | Lab | Lab |
|  |  | Pre-Treatment | Pre-Treatment | Post-Treatment |
|  |  | *Prepartum* | *Prepartum* | *Postpartum* |
| **AR (*n*=9)** |  |  |  |  |
|  | PSI |  |  | 7.44 (*SD*=5.46) |
|  | EPDS | 3.44 (*SD*=3.54) |  | 5.33 (*SD*=5.46) |
|  | MNWS |  | 9.33 (*SD*=9.46) | 9.33 (*SD*=9.46) |
|  | QSU-brief |  | 24.67 (*SD*=22.78) | 20.22 (*SD*=18.66) |
|  | TLFB |  |  | 3.63 (*SD*=16.80) |
|  | Breath CO |  | 4.50 (*SD*=7.84) | 2.25 (*SD*=2.71) |
|  |  |  |  |  |
| **Control (*n*=8)** |  |  |  |  |
|  | PSI |  |  | 4.37 (*SD*=1.51) |
|  | EPDS | 2.75 (*SD*=3.41) |  | 2.50 (*SD*=2.45) |
|  | MNWS |  | 8.75 (*SD*=6.92) | 4.25 (*SD*=4.27) |
|  | QSU-brief |  | 25.50 (*SD*=28.04) | 11.75 (*SD*=3.41) |
|  | TLFB |  |  | 9.00 (*SD*=16.81) |
|  | Breath CO |  | 1.88 (*SD*=4.55) | 2.43 (*SD*=4.79) |

Table Note: Descriptive statistics for laboratory data. Unless otherwise noted, n=9 (AR group) or n=8 (Control). Breath CO Visit 1: (AR: n=8; Control n=8); Breath CO Visit 2: (AR: n=8; Control n=7); TLFB: (AR: n=8; Control n=7). PSI = Items from Parental Stress Index; MNWS = Minnesota Nicotine Withdrawal Scale. EPDS= Edinburgh Postnatal Depression Scale. QSU-Brief = Questionnaire for Smoking Urges. TLFB = Reported Number (Sum) of Cigarettes on Timeline Followback. CO = Carbon Monoxide

Table S3: EMA Summary Statistics

| **Group ↓** | **Assessment ↓** | **All Assessments** | **Prepartum** | **Postpartum** |
| --- | --- | --- | --- | --- |
|  |  | ***N*=575** | ***n*=396** | ***n*=179** |
| **AR** |  |  |  |  |
|  | Attentional Bias (ms) | -47.53 (*SD*=141.83) | -52.78 (*SD*=143.81) | -37.89 (*SD*=141.70) |
|  | Craving (1-7) | 2.08 (*SD*=2.14) | 1.25 (*SD*=1.13) | 3.24 (*SD*=2.64) |
|  |  |  |  |  |
| **Control** |  |  |  |  |
|  | Attentional Bias (ms) | -0.01 (*SD*=83.28) | +0.50 (*SD*=86.11) | -2.81 (*SD*=66.74) |
|  | Craving (1-7) | 1.42 (*SD*=1.30) | 1.44 (*SD*=1.32) | 1.34 (*SD*=1.23) |
|  |  |  |  |  |

Table Note: 7 AR subjects and 7 Control subjects contributed EMA data. 6 AR subjects and 7 Control subjects contributed EMA data to the prepartum phase and 7 AR subjects and 4 Control subjects contributed EMA data to the postpartum phase. *n*=number of assessments. *n*s for each group for Craving: AR prepartum (*n*=170), AR postpartum (*n*=120). Control prepartum (*n*=226), Control postpartum (*n*=59); *n*s for each group for Parental Stress: AR prepartum (*n*=166), AR postpartum (*n*=118). Control prepartum (*n*=226), Control postpartum (*n*=58); *n*s for each group for Attentional Bias: AR prepartum (*n*=33), AR postpartum (*n*=18). Control prepartum (*n*=186), Control postpartum (*n*=34). *n*s for Control group are larger for attentional because bias scores can be computed on Control trainings and the VP assessment. In the Control group, attentional bias assessed in the Control trainings and VP assessment were of similar magnitude (*M*=-2.34 ms, *SD*=122.48 and *M*=0.79 ms, *SD*=65.23 respectively).

Table S4: Analyses of Lab Data

| **IV↓** | **DV ↓** | **Covariate** | ***n*** | ***df*** | ***F*** | ***p*** |
| --- | --- | --- | --- | --- | --- | --- |
|  |  |  |  |  |  |  |
| Group (AR vs Control) | PSI | (None) | 17 | 1, 15 | 2.36 | .15 |
| Group (AR vs Control) | Post-treatment EPDS | Pre-treatment EPDS | 16 | 1, 14 | 1.18 | .30 |
| Group (AR vs Control) | Post-treatment MNWS | Pre-treatment MNWS | 16 | 1, 14 | 0.69 | .42 |
| Group (AR vs Control) | Post-treatment QSU | Pre-treatment QSU | 16 | 1, 14 | 2.65 | .13 |
| Group (AR vs Control) | Post-treatment CO | Pre-treatment CO | 14 | 1, 12 | 0.32 | .58 |
|  |  |  |  |  |  |  |

Table Note: Data are results from ANOVA (No covariate) or ANCOVA. *n* =number of subjects (*n*s differ across analyses due to missing data). *F* values reflect tests of between-group (AR vs. Control) differences in lab measures assessed post-treatment (Study Completion) controlling for pre-treatment (Enrollment or Screening) data where possible


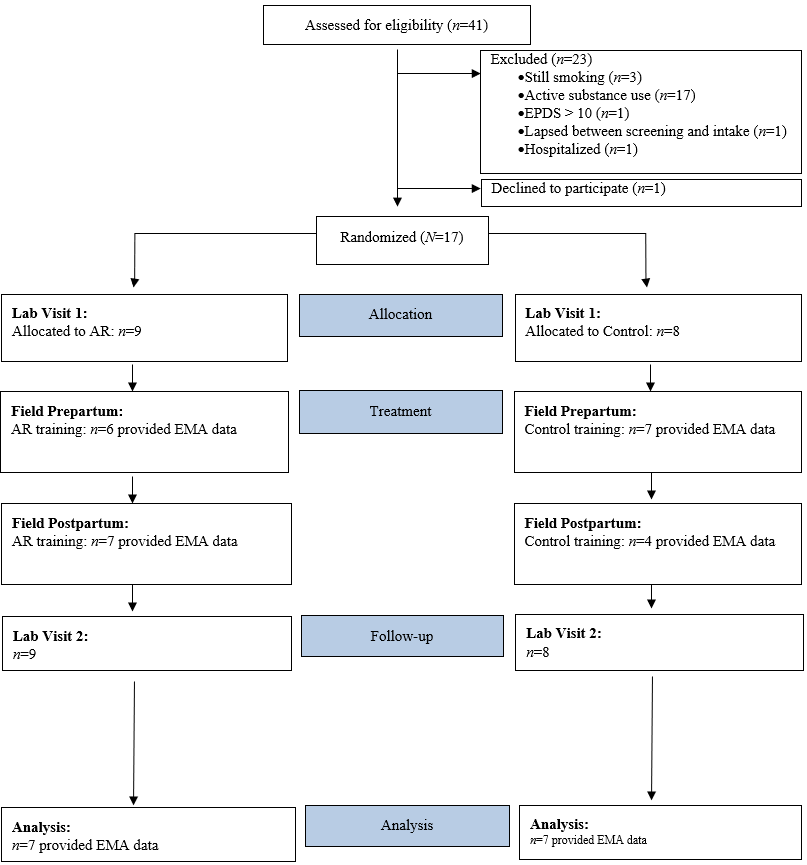


Figure S1. Consort Chart for Study
